# Chronic Progressive External Ophthalmoplegia (CPEO): Rehabilitation utilizing scleral contact lenses

**DOI:** 10.1016/j.ajoc.2025.102411

**Published:** 2025-08-21

**Authors:** Nir Erdinest, Nadav Shemesh, Naomi London, David Landau, Itay Lavy

**Affiliations:** a Department of Ophthalmology, Hadassah Medical Center, Faculty of Medicine, Hebrew University of Jerusalem, Israel; b Private Practice, Jerusalem, Israel

**Keywords:** External ophthalmoplegia, Chronic progressive external ophthalmoplegia (CPEO), Scleral contact lenses, Visual rehabilitation, Ocular surface disease

## Abstract

**Purpose:**

To evaluate the use of scleral contact lenses in managing symptoms of Chronic Progressive External Ophthalmoplegia (CPEO) after failed conventional treatments.

**Observations:**

A 69-year-old female with CPEO presented with persistent discomfort and blurry vision despite artificial tears and ointment treatments. Diagnosed with blepharoptosis, myogenic ptosis, dry eye syndrome, dermatochalasis, and lagophthalmos, she showed no improvement post-prolene frontalis suspension surgery. Scleral lenses provided immediate ptosis relief, improving spectacle-corrected visual acuity from 6/12 (−3) and 6/15 (−2) to 6/6 (−1) and 6/7.5 (−2) in the right and left eyes, respectively. Prior to scleral lens fitting, ocular surface staining showed an Oxford score of 3.0 in both eyes, which improved to <1 and remained stable through the four-year follow-up. The OSDI score decreased from 95.83 to 4.17, with sustained symptom relief and stable visual clarity reported throughout, without complications.

**Conclusions and importance:**

Scleral contact lenses provided significant relief from ptosis, markedly improved visual acuity, substantially reduced ocular surface damage, and nearly eliminated subjective dry eye symptoms in this complex CPEO case. These lenses are recommended as a primary therapeutic option for CPEO patients with ocular surface complications when conventional treatments are ineffective, offering sustained symptom relief and enhanced visual function.

## 1 Introduction

Chronic progressive external ophthalmoplegia (CPEO) is a rare mitochondrial disorders in adults.^1^, ^2^, ^3^ The clinical phenotype of CPEO characterized by external ophthalmoplegia, a gradual onset of ptosis (usually symmetric), high frequency sensorineural hearing loss (SNHL), and progressive dysphagia.^4^, ^5^, ^6^ To note, significant proptosis, or pupil involvement are not features of CPEO and such presentation should prompt evaluation for alternative etiologies.^1^^,^^4^, ^5^, ^6^ CPEO can also be associated with other neurological or systemic manifestations, such as proximal muscle weakness, ataxia and cardiac conduction defects, and it's severity and progression may vary significantly.

Mitochondrial DNA mutations are increasingly recognized as the etiology for CPEO syndromes; studies found that most CPEO cases are from a single heteroplasmic mitochondrial DNA deletion (ranging from 1.3 to 9.1 kilobases).^1^, ^2^, ^3^^,^^5^^,^^6^ The diagnosis of CPEO is based on clinical features, family history, and laboratory investigations, including serum creatine kinase, electromyography, muscle biopsy, and genetic testing. The management of CPEO is primarily supportive, with patients requiring close monitoring. Symptomatic treatments may include corrective lenses, ptosis surgery, strabismus surgery, and coenzyme Q10 supplementation. Genetic counseling is recommended for affected individuals and their families.^7^

One of the secondary complications in patients with CPEO is *exposure keratopathy,* resulting from lagophthalmos and impaired blinking due to levator and orbicularis weakness.^10^, ^11^, ^8^, ^9^ Exposure keratopathy is a condition in which the corneal surface is damaged due to prolonged or incomplete eyelid closure, leading to direct environmental exposure of the ocular surface. This results in progressive desiccation, inflammation, epithelial breakdown, and in severe cases, ulceration and scarring of the cornea.^8^^,^^9^^,^^12^^,^^13^

The condition most commonly arises from neuromuscular dysfunction, facial nerve palsy, or mechanical lagophthalmos, and it is exacerbated during sleep when Bell's phenomenon and blink reflexes are insufficient.^14^^,^^15^

Pathophysiologically, exposure disrupts tear film stability and increases tear evaporation, promoting a cascade of ocular surface damage, which starts with superficial punctate keratitis and potentially progresses to microbial keratitis or stromal melt.^10^^,^^11^^,^^16^^,^^17^ Management is tiered by severity and includes aggressive lubrication, nighttime eyelid taping, moisture goggles, punctal occlusion, botulinum-induced ptosis, temporary or permanent tarsorrhaphy, and increasingly, the use of scleral lenses, which protect and hydrate the corneal surface.^14^, ^15^, ^16^, ^17^ Early identification and individualized treatment strategies are crucial for preventing irreversible visual impairment.^10^^,^^11^^,^^16^^,^^17^

The present case describes a patient with CPEO who presented with severe ptosis, lagophthalmos, and dry eye syndrome primarily attributable to exposure keratopathy caused by incomplete blinking mechanisms. These persisted despite various medical and surgical interventions. Using scleral contact lenses proved to be an effective and non-invasive solution, highlighting their potential role in managing complex ocular surface disorders in neuromuscular syndromes.

## 2 Case presentation

A 69-year-old female presented with a chief complaint of discomfort in both eyes that increased as the day continued, as well as blurry vision. The patient visited several community ophthalmologists in 2018 with similar complaints, and instilled artificial tear drops and ointment at night. Until time (T) = -4M, (T = 0 time point in months defined as the initiation of scleral lens fitting; M = months), she would instill drops every 20–30 minutes, alternating between Systane Hydration (Alcon, USA) and Systane Gel Drops (Alcon, USA), experiencing minor relief for 5 minutes at a time. Furthermore, she spread a thin layer of Duratears (Alcon, Belgium) ointment at bedtime. The patient would meticulously try to control her environment using a humidifier and avoid surroundings with extremely low humidity, such as air-conditioned rooms whenever possible. At T = −4M, she presented via referral to the neuro-ophthalmology and oculoplastic units with a diagnosis of CPEO.

**Fig. 1:**
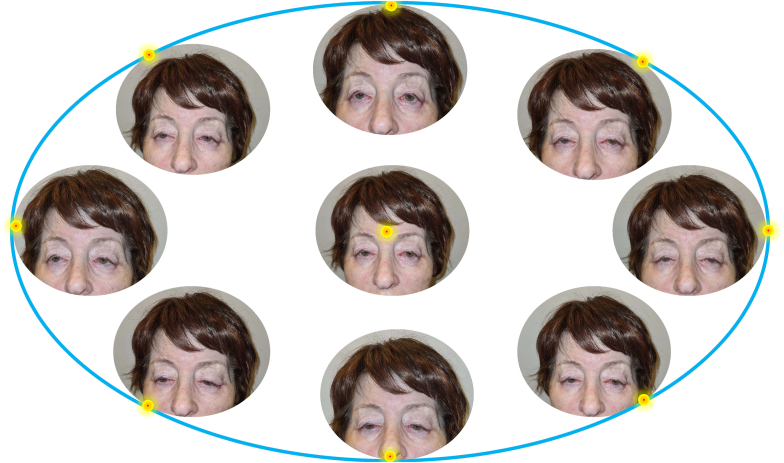
**Marked limitation of eye movements in all directions of endpoint gaze** The patient was asked to look at a target at the end points of gaze. A limitation of eye movements without diplopia was observed.

The patient was diagnosed with pupil-preserving blepharoptosis in both eyes, myogenic ptosis, dry eye syndrome, dermatochalasis, and lagophthalmos measured at 3.5mm and 2.5mm in the right and left eye, respectively. The extraocular motility assessment indicated a limited range of eye movement, approximately 1mm, leading the patient to compensate by extensive head movement and eyebrows elevation (Fig. 1).

A mixing of 1 % fluorescein strip (BioGlo, ophthalmic strips; Hub Pharmaceuticals, Scottsdale, Arizona, USA) and a drop 1 % lissamine green strip (GreenGlo; Hub Pharmaceuticals LLC, Rancho Cucamonga, California, USA) dissolved in one drop of saline (Isotonic saline solution 0.9 % NaCl, Braun, Melsungen AG, Melsungen, Germany). After the instillation of the dye into the cul-de-sac, the patient was instructed to blink twice, then she was evaluated immediately using a slit-lamp examination with a combination of a yellow filter and cobalt blue illumination. Two minutes after instillation, digital photographs of the eyes were taken using a conventional slit-lamp microscope (700GL; Takagi, Nagano, Japan) and SlitREC smartphone adapter (Digital Eye Center, Miami, Florida, USA) and smartphone (iPhone 11, Apple Inc, CA). The patient did not experience any significant ptosis improvement following a prolene frontalis suspension surgery in both eyes in T-3M. No amelioration of the ptosis was observed at the one-month and three-month post-operative follow-up appointments. Importantly, although the surgery failed to improve eyelid elevation or restore functional lid positioning, it did not lead to worsening of the patient's ocular surface condition. Eyelid closure remained incomplete but stable, and no new signs of exposure-related complications were observed during follow-up. Ocular surface staining, assessed using both Lissamine Green and Fluorescein dyes, yielded an Oxford score^18^ of 3.0 in both eyes, consistent with the preoperative findings. Meibomian gland dysfunction remained graded as 1 according to the Efron scale,^19^ and tear break-up time (TBUT) was consistently measured at 5 seconds in both eyes with a central breakup pattern. Furthermore, there was no subjective deterioration in visual clarity or ocular comfort reported by the patient. These findings indicate that while the blepharoplasty did not result in functional improvement, it also did not exacerbate the underlying dry eye disease or compromise ocular surface integrity.

At the three-month post-operative visit, two examinations were conducted six weeks apart, the patient exhibited persistent signs of dry eye, including superficial punctate keratitis, ocular surface staining with an Oxford score of 3.0 in both eyes using Lissamine Green and Fluorescein dyes, meibomian gland dysfunction graded as 1 according to the Efron scale, tear break-up time (TBUT) of 5 seconds in both eyes with central breakup pattern, and mild conjunctival hyperemia. She continued to rely on the regimen of two types of Systane eye drops during the day and VitA-POS ointment at night but without any relief from her symptoms.

**Fig. 2:**
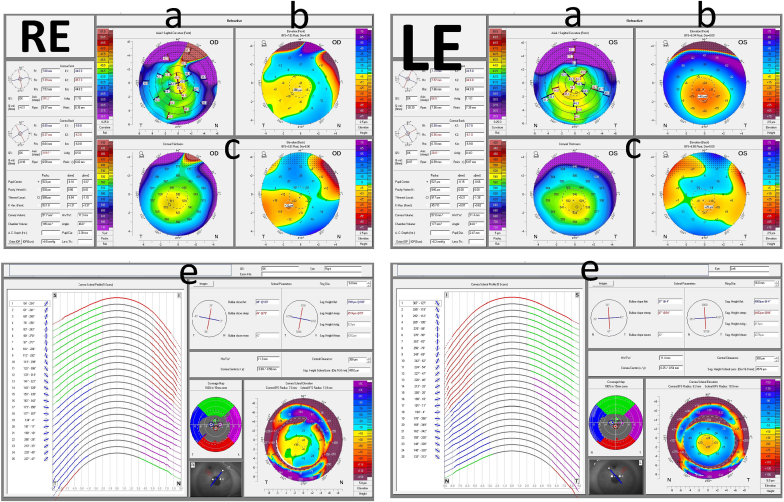
**Corneal topography, including Cornea Scleral Profile (CSP) of the patient.** a. Anterior sagittal mapping b. Anterior elevation c. anterior and posterior elevation d. corneal pachymetry e. Cornea Scleral Profile (CSP) mapping.

The refractive error was +10.75/-0.75 × 144° in the right eye and +10.75/-0.50 × 106° in the left. The BCVA with single-focal spectacles was 6/12 (20/40) in the right eye and 6/15 (20/50) in the left eye, with binocular BCVA of 6/12 (20/40). Corneal topographic analysis revealed regular astigmatism of 1.7 diopters in the right cornea and irregular astigmatism of 1.1 diopters in the left cornea, respectively (Fig. 2).

A complete eye examination was performed, including a corneoscleral topographic analysis with the Scheimpflug camera-based system Pentacam (Oculus Optikgerate GmbH, Wetzlar, Germany) and the Pentacam cornea scleral profile (CSP) model. The CSP scan is a tear film-independent measurement with automatic release and utilizing to assist the fitting of asymmetric scleral contact lenses based on corneoscleral toricity (Fig. 2e).

Patient's K max, minimum pachymetry and max anterior elevation (3mm of center diameter) were 53.1D, 508μ,10μ and 45.7D, 517μ, 40μ for the right and the left eye, respectively. The inferior–superior (I–S), Keratoconus index (KI) and Keratoconus percentage index (KISA%) values were 1.8, 3.69, 1.25, and 2.80, 61.045, 6.536 for the right and left eye respectively.

Prior to scleral lens fitting, slit lamp examination revealed bilateral diffuse superficial punctate keratitis (SPKs), predominantly involving the inferior third of the cornea. Ocular surface staining according to the Oxford Grading Schema, yielding a score of 3.0 in each eye.

Both eyes demonstrated meibomian gland dysfunction (MGD) of Grade 1, limbal hyperemia of Grade 1, and no evidence of corneal neovascularization (Grade 0) or papillary conjunctivitis (Grade <1). In addition, mild conjunctival redness grade 2 and conjunctivochalasis were also noted in both eyes, along with a reduced tear meniscus height of approximately 0.1 mm. The ocular surface status was graded using the Efron Grading Scales.

In T = 0, the patient attempted an Advance Vision Technology (AVT) scleral lens of 22 mm diameter (Advance Vision Technology, Lakewood, Colorado, USA), but complained of difficulty handling the scleral lenses, particularly insertion and removal. In addition, the patient attempted an UltraHealth hybrid contact lens (SynergEyes, Inc., Carlsbad, CA) but complained of difficulty handling, particularly their removal.

**Fig. 3:**
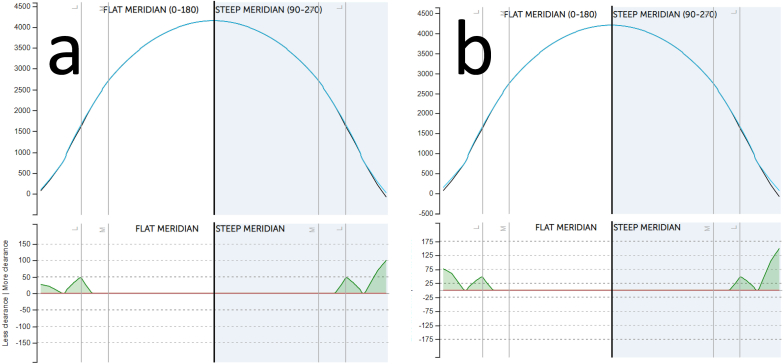
The fitting profile of the scleral lenses. Profile a and b represent the right and left eye, respectively.

After the patient handling difficulties, fitting a Onefit MED Blanchard 16.40mm (Blanchard Contact Lens Inc., Sherbrooke, Quebec, Canada (was tried in T = 0. Before scleral contact lens fitting, an endothelial cell count was performed and measured above 1000 cells/mm, which is acceptable for scleral lens wear.^20^ This threshold is critical because a cell count below 1000 cells/mm^2^ may increase the risk of corneal edema during lens wear due to reduced oxygen transmissibility through the lens. Several scleral profilometers helped to identify the scleral structures and design better asymmetric periphery lenses. Profilometry played a key role in mapping the scleral structures, enabling the design of asymmetric periphery lenses that conform accurately to the patient's unique ocular topography.The power of the scleral contact lenses was +9.50/-0.75x150, +9.00/-0.50x100 for the right and left eye, and the complete fitting details are shown in Table 1 and Fig. 3.

**Table 1:** Scleral contact lens properties.

Properties	Right contact lens data	Left contact lens data
Contact lens raw material (Diameter)	Optimum Infinite (16.4 mm)	Optimum Infinite (16.4 mm)
SAG (Limbus)	4150μ (+50μ)	4200μ (+75μ)
**EDGE (Toric Haptic)**		
Flat Meridian (Steep Meridian)	+100μ (+25μ)	+150μ (+75μ)
Power (D)	+9.50/-0.75x150	+9.00/-0.50x100
Contact lens center thickness	430μ	420μ
Estimate Dk/t	21	20

SAG: Sagittal Height/Depth, Measured in microns (μm); CCR: Central Curve Radius; D: Diopters; Dk: Diffusion coefficient, oxygen solubility coefficient expressed as (cm^2^/sec)(mL O_2_/mL x mm Hg).

**Fig. 4:**
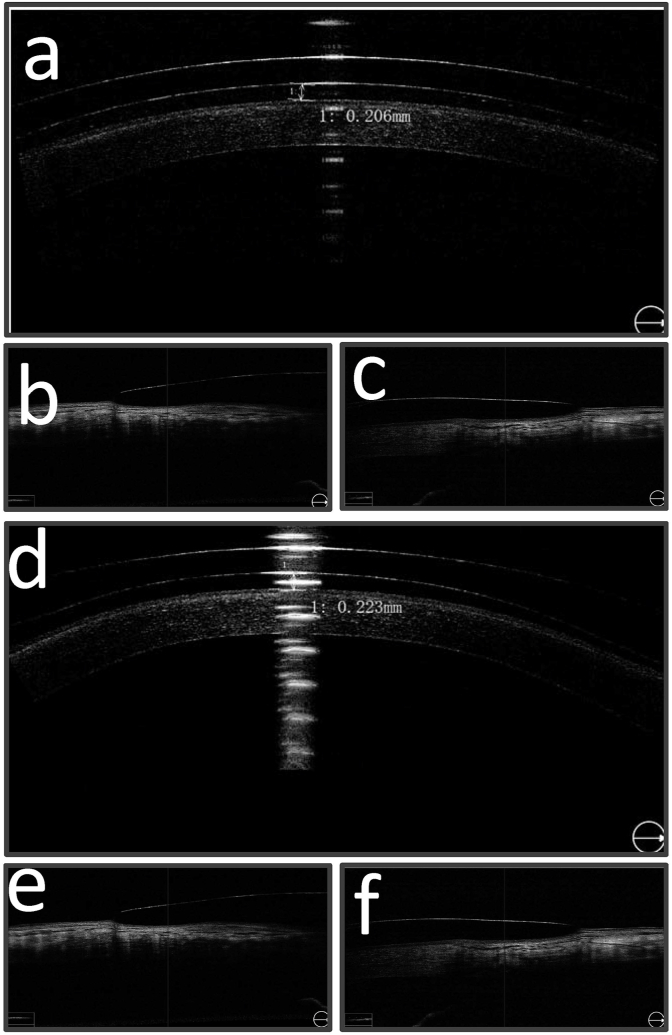
OCT examination a. OCT measurements of scleral contact lens show 206μ and 223μ apical clearance after 12 hours of contact lens wear in the right eye (a) and left eye (d), respectively. The landing zone of the scleral contact lenses aligns with the conjunctiva, exerting no pressure on either the right eye (b and c) or the left eye (e and f).

**Fig. 5:**
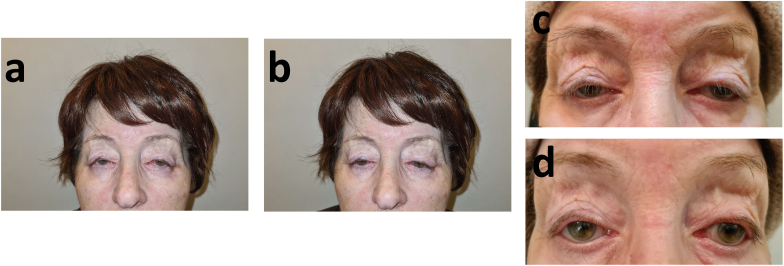
The patient in a natural pose (a), the patient was asked to close her eyes (b). The patient before placing the scleral lenses on the eyes demonstrates bilateral ptosis of with a Margin to Reflex Distance (MRD) of less than 0.5 mm in the right eye and an MRD of 0.5 mm in the left eye (c). After four years of scleral lens wear, the value of 4.2 mm MRD was preserved (d).

Contact lens fitting was evaluated with the slit lamp and optical coherence tomography (OCT) (see Fig. 4). The OCT demonstrated acceptable clearance of 206μ and 223μ apical clearance after 12 hours of contact lens wear in the right eye (Fig. 5a) and left eye (Fig. 5d), respectively.

The slit lamp demonstrated acceptable clearance in the limbal zone and the scleral edge with conjunctival blanching. The scleral edge aligns to the conjunctiva without any pressure in the right eye (Fig. 5b and c) and left eye (Fig. 5e and f).

The scleral contact lens exhibited excellent centration. Following the quadratic scleral lens fitting, the patient's best corrected visual acuity improved to 6/7.5 (+1) and 6/7.5 (−2) in the right and left eye, respectively. The patient's near acuity improved to Jaeger 1 with +2.75D reading spectacles worn over the contact lens.

The patient achieved improved visual acuity and comfort. The scleral lens with quadrant asymmetric peripheral design was well tolerated for 14–15 hours of daily wear (refreshing with non-preserved saline every 4–5 hours) without complaints or complications. The patient cleaned the scleral lenses with Boston Advance Cleaner and Boston Advance Comfort Formula conditioning solution (Bausch + Lomb, Inc. Rochester, NY, USA) and disinfected the lenses using Ever Clean plus 3 % hydrogen peroxide solution (Avizor Alvera, Avizor SA, Madrid, Spain).

**Fig. 6:**
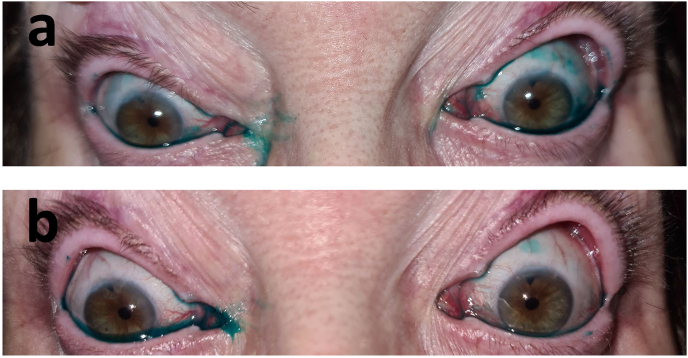
Lissamine green staining in the patient before treatment with scleral contact lenses. a. Photograph of lissamine green staining pre-scleral lens (T = 0) fitting b. Photograph of lissamine green staining four years (T = +48) after scleral lens fitting (the scleral lenses were removed 10 minutes before the photograph was taken). Both eyes' picture was recorded by smartphone (iPhone 12 Pro, Apple Inc, California, USA) 2 min after 1 % lissamine green instillation.

The patient returned for follow-up visits one week, one month, three months, six months and one year after dispensing. Visual acuity was stable, and fit assessment continued to show corneal vaulting with no pressure on the scar or excessive pressure on the other ocular surface tissue. Fluorescein and lissamine green staining were performed to detect and evaluate conjunctival and corneal staining (Fig. 6). In all follow-up visits, the ocular health was intact, and there was no evidence of conjunctival or corneal staining apart from the one week aftercare visit. Furthermore, there were no limbal or bulbar hyperemia, tarsal giant papillary conjunctivitis, or discharge and debris on the lens in any follow-up visit.

### 2.1 Aftercare and follow-up

Post-dispensing evaluation revealed progressive clinical improvement characterized by enhanced ocular surface stability and reduced inflammatory signs. The initial one-week assessment demonstrated a favorable therapeutic response with bilateral improvement in SPKs and ocular surface staining achieving subclinical levels according to the Oxford grading schema. Efron grading assessment revealed well-controlled meibomian gland dysfunction, limbal hyperemia, and papillary conjunctivitis at minimal grades, with no evidence of corneal neovascularization throughout the follow-up period.

Extended monitoring over 48 months confirmed sustained treatment efficacy and continued clinical stability. Oxford grading consistently demonstrated maintained ocular surface health below clinical thresholds, whereas Efron classification revealed persistent but stable low-grade inflammatory changes, reflecting the underlying chronic nature of the condition. Tear film break-up time showed minor fluctuations during the later follow-up phases, along with conjunctival changes including mild hyperemia and conjunctivochalasis. Visual acuity and near vision remained stable throughout the follow-up period.

A comprehensive longitudinal analysis of patient-reported outcomes using the Ocular Surface Disease Index revealed marked symptomatic improvement from baseline, with initial severe symptom scores decreasing dramatically within the first six months of treatment. Despite minor fluctuations during the extended follow-up period, symptom scores remained consistently below baseline levels (Table 2).

**Table 2:** Integrated timeline of diagnostic findings and outcomes.

Time Point	Clinical Event	Diagnostics and Clinical Findings	OSDI Score
T = -3M and -4M	Pre-fitting evaluation	Dry eye, SPKs, ocular surface staining (Oxford score of 3.0 OU), TBUT of 5 sec, tear meniscus ∼0.1 mm. MGD Grade 1, limbal hyperemia Grade 1, no neovascularization, papillary conjunctivitis <1. SE +10.375 D OU. BCVA 6/12 OD, 6/15 OS.	95.83
T = 0	Scleral lens fitting initiated	Pentacam CSP-guided. ECC >1000/mm^2^. Final lens: SAG 4150 μm (OD), 4200 μm (OS); SE: +9.1 D OD, +8.75 D OS.	95.83
T = +1W	First follow-up	Marked reduction in SPK area and density. Oxford score <1 OU. TBUT remained at 5 sec OU. Tear meniscus ∼0.1 mm. MGD Grade 1, limbal hyperemia Grade 1, papillary conjunctivitis <1. Mild conjunctival redness and conjunctivochalasis, Grade 2. BCVA: 6/7.5 (+1) OD, 6/7.5 (−2) OS. Near vision: J1 with +2.75D.	16.67
T = +6M	Second follow-up	Clinical stability. Oxford score <1 OU. TBUT 5 sec OU. Tear meniscus ∼0.1 mm. MGD Grade 1. Papillary conjunctivitis <1. Mild conjunctival redness and conjunctivochalasis, Grade 1. BCVA: 6/7.5 (+1) OD, 6/7.5 (−2) OS. Near vision: J1 with +2.75D.	4.17
T = +12M	One-year follow-up	No progression. Oxford score <1 OU. TBUT 5 sec OU. Tear meniscus ∼0.1 mm. MGD Grade 1. Papillary conjunctivitis <1. Mild conjunctival redness and conjunctivochalasis, Grade 1. BCVA: 6/7.5 (+1) OD, 6/7.5 (−2) OS. Near vision: J1 with +2.75D.	6.25
T = +24M to +48M	Long-term follow-up	Continued surface stability. Oxford score <1 OU. TBUT decreased to 4 sec OU. Tear meniscus ∼0.1 mm. MGD Grade 1, limbal hyperemia Grade 1. Papillary conjunctivitis increased to Grade 1. Mild conjunctival redness and conjunctivochalasis, Grade 1. BCVA: 6/7.5 (+1) OD, 6/7.5 (−2) OS. Near: J1 with +2.75D.	10.42 (T+24), 8.33 (T+36), 10.42 (T+48)

T: time point, W: week, M: months, T = 0 was defined as the initiation of scleral lens fitting, OSDI: Ocular Surface Disease Index, SPK: superficial punctate keratitis, TBUT: tear break-up time, SE: spherical equivalent, VA: visual acuity, BCVA: best corrected visual acuity, OU: oculus uterque (both eyes), OD: oculus dexter (right eye), OS: oculus sinister (left eye), ECC: endothelial cell count, CSP: corneoscleral profile, MGD: meibomian gland dysfunction, OCT: optical coherence tomography, D: diopters, SAG: sagittal height/depth, Dk/t: oxygen transmissibility, J1: Jaeger near visual acuity level 1, I–S: inferior–superior index, KI: keratoconus index, KISA%: keratoconus percentage index.

## 3 Discussion

The present case describes a patient with CPEO who presented with severe ptosis, lagophthalmos, and dry eye syndrome primarily attributable to exposure keratopathy caused by incomplete blinking mechanisms. Despite frontalis suspension surgery, lagophthalmos persisted, exacerbating the exposure keratopathy and ocular surface symptoms. These manifestations proved refractory to conventional interventions, but scleral contact lenses provided an effective solution, demonstrating significant potential for managing this complex case. The current case report demonstrates effective management of a challenging CPEO through scleral contact lenses. Scleral lenses have become an increasingly popular treatment option for ocular surface disorders, including severe dry eye, corneal irregularities, and complications following refractive surgery. The lenses provide a stable, tear-filled reservoir that protects the cornea and improves visual acuity, while reducing discomfort associated with ocular surface disease.

The patient in this case study presented with a history of CPEO and dry eyes related problems, including discomfort and blurred vision. Despite previous treatments with artificial tears and ointments, as well as surgical interventions like prolene frontalis suspension and therapeutic lenses, the patient's condition remained challenging to manage.

Contact lens wear and severe ocular surface disease can be challenging to manage, as dry eye symptoms can worsen with lens wear, and ocular surface disease can compromise the fit and comfort of contact lenses. However, in this case, scleral contact lenses fitting successfully addressed the patient's ocular surface problem and improved her visual function.

Scleral lenses are particularly effective in managing severe dry eye disease. The large diameter of scleral lenses allows them to vault over the cornea and limbus, creating a fluid-filled reservoir between the lens's posterior surface and the cornea's anterior surface. This fluid reservoir helps to hydrate the cornea and conjunctiva, reducing symptoms of dryness. Additionally, scleral lenses can be filled with preservative-free saline solutions or viscous comfort drops, further enhancing the ocular surface environment.^21^ The scleral lens material selection was based on the patient's high positive power prescription due to aphakia. The oxygen transmissibility (Dk) was calculated using the lens manufacturer's formula, which accounts for lens vaulting, lens thickness, and tear layer thickness, as follows: (1/(Lens thickness/Material Dk) + (Tear thickness/80)) ∗ 100. Over-refraction performed on the trial lens determined the lens thickness used in this formula to optimize fit and oxygen delivery to the cornea. Based on the oxygen transmissibility (Dk) calculated, scleral lens materials like Optimum Infinite would have an oxygen transmissibility of 21 Dk.^22^^,^^23^ Given the suboptimal oxygen levels, the patient was advised to remove the lenses every 3 hours and refresh them, a process that takes approximately 5 min.

CPEO is a rare mitochondrial disorder primarily observed in adults, characterized by gradual external ophthalmoplegia, symmetric ptosis, sensorineural hearing loss, and progressive dysphagia.^11^ The etiology is linked to mitochondrial DNA mutations, particularly a single heteroplasmic deletion.^11^ CPEO can present as pure CPEO or a CPEO "plus" syndrome characterized by additional multisystem features. Clinical features include progressive ptosis, ophthalmoplegia, diplopia, and blurred vision, which may be associated with other neurological or systemic symptoms. The pathophysiology involves mitochondrial dysfunction in the extraocular muscles, leading to weakness and atrophy. CPEO is a complex disorder with various underlying mechanisms, including oxidative stress, calcium dysregulation, and impaired mitochondrial dynamics.^11^

While the case study highlights the complexity of diagnosing and treating the ophthalmic manifestations of CPEO, it also demonstrates the potential of scleral contact lenses as a therapeutic option. The patient's significant improvement in visual acuity and ocular comfort after successfully fitting a semi-scleral contact lens underscores the utility of this modality in addressing the corneal irregularities and ocular surface issues commonly encountered in CPEO. The progression of the patient's OSDI scores further illustrates the subjective beneficial impact of the scleral lens intervention over time.

## 4 Conclusions

Scleral contact lenses played a crucial role by addressing the patient's ptosis, improving visual acuity beyond other optical modalities, and facilitating the healing of dry eye-affected surfaces. With their asymmetric peripheral design, these lenses provided a stable and effective solution for the patient's complex ocular condition, sustained for at least four years.

## CRediT authorship contribution statement

**Nir Erdinest:** Writing – review & editing, Formal analysis, Data curation, Conceptualization. **Nadav Shemesh:** Writing – review & editing, Formal analysis, Data curation. **Naomi London:** Writing – review & editing, Writing – original draft, Formal analysis. **David Landau:** Writing – review & editing, Supervision, Formal analysis, Data curation. **Itay Lavy:** Writing – review & editing, Formal analysis, Data curation, Conceptualization.

## Patient consent

This report does not contain any personal information that could lead to the identification of the patient.

## Authorship

All authors attest that they meet the current ICMJE criteria for Authorship.

## Research ethics

We further confirm that any aspect of the work covered in this manuscript that has involved human patients has been conducted with the ethical approval of all relevant bodies and that such approvals are acknowledged within the manuscript.

## Statement of ethics

Ethical approval is not required for this study in accordance with local or national guidelines.

## Funding support

No funding or grant support.

## Declaration of competing interest

The authors declare that they have no known competing financial interests or personal relationships that could have appeared to influence the work reported in this paper.
